# Protective association between recent insecticide-treated bed net use and falciparum malaria in three provinces of the Democratic Republic of the Congo

**DOI:** 10.64898/2026.09.15.26363108

**Published:** 2026-09-16

**Authors:** Danielle L. Wiener, Eddy Kieto, Melchior M. Kashamuka, Mvuama Nono, Jonathan J. Juliano, Fernandine Phanzu, Adrien N’Siala, Tommy Nseka, Antoinette K. Tshefu, Albert Kalonji, Tim Sheahan, Joris Likwela, Jonathan B. Parr

## Abstract

Recent research highlights threats to the effectiveness of insecticide-treated bed nets (ITNs) against malaria transmission. This cross-sectional study (N=2819) investigated associations between bed net usage and malaria rapid diagnostic test (RDT) positivity among those presenting with malaria symptoms in three provinces of the Democratic Republic of the Congo (DRC). A log-binomial regression model was used to determine RDT-confirmed malaria prevalence ratios; prior malaria diagnosis and location of care were assessed as modifiers. Using a bed net the prior night was associated with lower risk of malaria diagnosis [PR of 1.39 (95% CI: 1.20, 1.62), with a quantitative bias analysis adjusted result of PR = 1.64]. ITN use reduced risk of malaria diagnoses regardless of prior malaria infection. However, prior diagnosis did modify the relationship between bed net usage and malaria RDT positivity, with prior diagnosis reducing bed net effectiveness. These findings add to existing literature showing the benefits of ITN use in highly malaria-endemic settings like the DRC.

## Full Text Availability

The license terms selected by the author(s) for this preprint version do not permit archiving in PMC. The full text is available from the preprint server.

